# Real-world predictors of survival in patients with limited-stage small-cell lung cancer in Manitoba, Canada

**DOI:** 10.3389/fonc.2023.1191920

**Published:** 2023-12-06

**Authors:** David E. Dawe, Rebekah Rittberg, Iqra Syed, Mary Kate Shanahan, Daniel Moldaver, Oliver Bucher, Katie Galloway, Kayla Reynolds, James T. Paul, Craig Harlos, Julian O. Kim, Shantanu Banerji

**Affiliations:** ^1^ Department of Internal Medicine, University of Manitoba, Winnipeg, MB, Canada; ^2^ Department of Hematology and Medical Oncology, CancerCare Manitoba, Winnipeg, MB, Canada; ^3^ CancerCare Manitoba Research Institute, CancerCare Manitoba, Winnipeg, MB, Canada; ^4^ AstraZeneca Canada, Mississauga, ON, Canada; ^5^ Department of Epidemiology and Cancer Registry, CancerCare Manitoba, Winnipeg, MB, Canada; ^6^ Department of Cellular & Physiological Sciences, University of British Columbia, Vancouver, BC, Canada; ^7^ Department of Radiology, University of Manitoba, Winnipeg, MB, Canada; ^8^ Department of Radiation Oncology, CancerCare Manitoba, Winnipeg, MB, Canada

**Keywords:** small-cell lung cancer, limited-stage, performance status, real world, long-term survival

## Abstract

**Background:**

Although therapy for limited-stage small-cell lung cancer (LS-SCLC) is administered with curative intent, most patients relapse and eventually die of recurrent disease. Chemotherapy (CT) with concurrent radiotherapy (RT) remains the standard of care for LS-SCLC; however, this could evolve in the near future. Therefore, understanding the current prognostic factors associated with survival is essential.

**Objective:**

This real-world analysis examines factors associated with long-term survival in patients with LS-SCLC treated with CT in Manitoba, Canada.

**Methods:**

A retrospective cohort study was conducted using Manitoba Cancer Registry and CancerCare Manitoba records. Eligible patients were aged >18 years and had cytologically confirmed LS-SCLC diagnosed between January 1, 2004, and December 31, 2018, for which they received CT ± RT. Baseline patient, disease, and treatment characteristics and survival duration, characterized as short (<6 months), medium (6−24 months), and long term (>24 months), were extracted. Overall survival (OS) was estimated at one, two, and five years and assessed using Kaplan-Meier methods and Cox proportional hazards models.

**Results:**

Over the 15-year study period, 304 patients met the eligibility criteria. Long-term survivors comprised 39.1% of the cohort; at diagnosis, this subgroup was younger, more likely to have Eastern Cooperative Oncology Group Performance Status (ECOG PS) 0, and have normal lactate dehydrogenase, sodium, and hemoglobin levels. OS estimates for the entire cohort at one, two, and five years were 66%, 38%, and 18%, respectively. In the ECOG PS 0 subgroup, OS estimates at one, two, and five years were 85%, 52%, and 24%, respectively; OS estimates were 60%, 35%, and 17%, respectively, for ECOG PS 1−2 and were 47%, 23%, and 10%, respectively, for ECOG PS 3−4. OS was significantly higher among patients with normal serum sodium and hemoglobin levels than those with abnormal levels. Univariable hazard regression models found that ECOG PS, age at diagnosis, receipt of prophylactic cranial irradiation (PCI), and thoracic RT were associated with survival. On multivariable hazard regression, ECOG PS and receipt of PCI were associated with survival.

**Conclusion:**

Survival for greater than two years in patients with LS-SCLC treated with CT ± RT was associated with ECOG PS and receipt of PCI.

## 1 Introduction

Small-cell lung cancer (SCLC) is an aggressive disease typically featuring rapid growth and early development of locoregional and distant metastases (1, 2), which represents approximately 12% of all lung cancers in Canada (3). SCLC is classified according to disease extent as either limited stage (LS), which corresponds to most patients with 8th edition TNM stage I-IIIB, or extensive stage (ES), which corresponds to most patients with stage IIIC/IV (4). LS-SCLC refers to the presence of a tumor limited to one hemithorax, which historically corresponded to a 10 x 10 cm radiation treatment field, and accounts for approximately one-third of patients with SCLC (3, 5). An estimated 20% of patients with LS-SCLC survive to two years from diagnosis, and 10%–13% survive past five years (5–9). With treatment, median survival of patients with LS-SCLC is estimated at 14–20 months, while those who do not receive treatment typically survive for approximately 10–12 weeks (5, 7, 9, 10).

Therapy for LS-SCLC is administered with curative intent (11–14). The standard of care for LS-SCLC is platinum-based chemotherapy (CT) and concurrent radiotherapy (RT) (15, 16). CT is with either cisplatin or carboplatin, often in combination with etoposide (15, 16). Surgical resection may be considered for patients with T1-T2, N0, M0 (stage I) and no pathologic mediastinal involvement (17, 18). In patients with LS-SCLC who respond well to initial CT ± RT, prophylactic cranial irradiation (PCI) may be used to decrease the risk of developing brain metastases and to increase OS (14, 19). The use of PCI versus monitoring with MRI is currently being further evaluated in the MAVERICK (SWOG S1827) trial (20).

Despite a high level of initial response to initial therapy, most patients with LS-SCLC will relapse and eventually die of recurrent disease (9, 21, 22). Relapsed LS-SCLC is associated with a poor prognosis, and those with disease relapses within 6 months of completing their initial course of CT have less of a chance of responding to additional CT (9, 23). The existence of long-term LS-SCLC survivors has been recognized; however, patient, disease, and treatment characteristics associated with long-term survival are not well defined (9, 22).

Research in SCLC is ongoing and new therapeutic options may be available in the near future. Ongoing clinical trials are evaluating the safety and efficacy of immune checkpoint inhibitors (ICIs) with or following chemoradiotherapy (CRT) in patients with LS-SCLC (24, 25). To date, ICIs in combination with CT ± RT have been demonstrated to provide clinical benefit in patients with ES-SCLC and non-SCLC (NSCLC) (26–31).

Understanding the impact of baseline patient characteristics on choice of treatment and clinical outcomes is essential for improving outcomes in patients with LS-SCLC. To date, few population-based studies have comprehensively examined the patient characteristics and treatment patterns associated with long-term survival among patients with LS-SCLC in Canada (32–34). Our group previously evaluated the effect of cisplatin vs. carboplatin on clinical outcomes of patients with ES-SCLC and LS-SCLC in a cohort of patients from CancerCare Manitoba (CCMB) (33). Patients treated with carboplatin (26.2% of the cohort) were more likely to have Eastern Cooperative Oncology Group Performance Status (ECOG PS) 3−4, elevated lactate dehydrogenase (LDH), and ES-SCLC than those receiving cisplatin. Unadjusted median overall survival (OS) was 224 vs. 322 days in the carboplatin and cisplatin groups, respectively (33). A separate analysis of the same cohort examined the impact of inpatient vs. outpatient CT administration on outcomes in a mixed group of patients with LS-SCLC and ES-SCLC (34). The majority (65.5%) of patients with LS-SCLC were ECOG PS 0, and 91.7% received outpatient CT ± RT. Multivariable analysis identified ECOG PS as an independent predictor of outcome. The two- and five-year OS estimates for the LS-SCLC cohort were 33.6% and 15.9%, respectively, and OS at two years was higher among outpatients (34.7% vs. 22.2%), but five-year OS was similar among inpatients and outpatients (16.7% vs. 15.8%) (34).

The present study expands on our earlier real-world retrospective cohort (33, 34), with additional data from patients diagnosed up to the year 2018. The objective of this analysis was to describe the characteristics and treatment regimens of patients with LS-SCLC who received CT ± RT in Manitoba, Canada, and to estimate the probability of OS for these patients to five years from diagnosis. Data were stratified by length of survival: short- (<6 months), medium- (6-24 months), and long-term (>24 months).

## 2 Materials and methods

### 2.1 Study design

This is a retrospective, population-based, cohort study of patients with LS-SCLC and treated with CT ± RT in the Canadian province of Manitoba, which has a catchment population of approximately 1.4 million universally insured persons with a single-source, publicly administered, healthcare system. This study was approved by the University of Manitoba Health Research Ethics Board (HREB H2015:154 [HS18575], RRIC #2015-31).

### 2.2 Study cohort

Eligible patients 1) were aged >18 years, 2) had cytologically confirmed LS-SCLC, and 3) received cytotoxic CT ± RT. Patients were excluded from the analysis if they did not receive CT, had NSCLC, or had ES-SCLC.

### 2.3 Data source

Data were obtained from a previously described study cohort from the Manitoba Cancer Registry (MCR) (34). The MCR is among the oldest cancer registries in North America and is operated by CCMB to collect, classify, and maintain detailed information on all cancer cases in Manitoba. Eligible patients in the initial study were diagnosed between January 1, 2004, and December 31, 2013, which was expanded for the current study to include patients diagnosed between January 1, 2014, and December 31, 2018. A manual review of all patients’ CCMB outpatient electronic medical records provided additional case details. Follow-up data were available until September 30, 2021.

### 2.4 Outcome measures

Outcome measures were descriptive characteristics of the patient cohort and the treatment regimens they received. Patient characteristics included current age, age at diagnosis, sex, smoking status, stage of disease at diagnosis, laboratory test results at diagnosis, and ECOG PS at diagnosis. Key parameters of laboratory testing included levels of LDH, sodium, and hemoglobin, which have been identified as important prognostic factors in LS-SCLC (35–38). Laboratory values in our database were acquired prior to cycle 1 and CT typically starts within 1-2 weeks of clinician assessment. If ECOG PS was not explicitly stated in the electronic medical record, it was derived from the description of patient functional status in the initial history and physical examination. Treatment characteristics included regimen received, such as CT (cisplatin or carboplatin and etoposide), RT, thoracic RT to lung or mediastinum (concurrent, sequential, or palliative), or brain RT (PCI or whole-brain RT). Of note, no patients in this cohort received ICIs since they were not available for SCLC patients, regardless of stage, during this period. We included patients classified as limited stage based on best understanding at the time of treatment administration, whereas Collaborative Stage (stage I-IV in **Table 1**) is determined retrospectively, which sometimes leads to upstaging.

**Table 1:** Baseline patient and disease characteristics.

Characteristic	Short- term survival	Medium- term survival	Long- term survival	*P*-value^a^
**Patients, n (%)** ^b^	29 (9.5)	156 (51.3)	119 (39.1)	
**Age, years, median (range)**	71 (54-85)	68 (35-87)	66 (45-90)	0.029^c^
**Sex, n (%)**				0.440^d^
**Male**	14 (48.3)	69 (44.2)	45 (37.8)	
** Female**	15 (51.7)	87 (55.8)	74 (62.2)	
**ECOG PS, n (%)**				0.003
**0−2**	22 (75.9)	139 (89.1)	111 (93.3)	
**3−4**	7 (24.1)	16 (10.3)	7 (5.9)	
**Collaborative stage, n (%)**				0.066
**I-I**I	−^e^	33 (21.2)	42 (35.3)	
**III-IV**	−^e^	119 (76.3)	77 (64.7)	
**Smoking status, n (%)**				0.271
**Never/prior smoking/unknown**	17 (58.6)	95 (60.9)	76 (63.9)	
**Current**	12 (41.4)	61 (39.1)	43 (36.1)	
**Smoking (pack years), median (range)**	45 (6-63)	40 (0-100)	40 (0-100)	0.1321^c^
**LDH,**^f^ **n (%)**				0.011
**Normal**	13 (44.8)	112 (71.8)	93 (78.2)	
**Elevated**	12 (41.4)	34 (21.8)	19 (16.0)	
**Sodium,**^g^ **n (%)**				0.013
**Normal**	22 (75.9)	125 (80.1)	108 (90.8)	
**Abnormal**	−^e^	29 (18.6)	11 (9.2)	
**Hemoglobin,**^h^ **n (%)**				0.008
**Normal**	16 (55.2)	88 (56.4)	84 (70.6)	
**Low**	11 (37.9)	67 (43.0)	35 (29.4)	
**T-Stage**				0.117
**T0/1**	-^e^	32 (20.5)	30 (25.2)	
**T2**	10 (34.5)	48 (30.8)	44 (37.0)	
**T3**	-^e^	17 (10.9)	16 (13.5)	
**T4/X**	11 (37.9)	59 (37.8)	29 (24.4)	
**N-Stage**				0.104
**N0**	-^e^	24 (15.4)	32 (26.9)	
**N1**	-^e^	21 (13.5)	19 (16.0)	
**N2/3/X**	22 (75.9)	110 (70.6)	68 (59.1)	

a Fisher exact test P-value;

b unknown data comprise the differences in characteristic subtotals and the group totals;

c Kruskall Wallis test P-value;

d Chi-square P-value;

e patient numbers ≤5 are censored based on requirements from Manitoba Health;

f elevated LDH: >230 U/L;

g abnormal sodium: <135 or >147 mEq/L;

h low hemoglobin: males <140 g/L, females <120 g/L. ECOG PS, Eastern Cooperative Oncology Group Performance Status; LDH, lactate dehydrogenase.

Clinical outcomes included treatment response and OS, which was defined as the time interval (months) from date of first CT treatment to date of death, censoring due to loss to follow-up, or end of the follow-up period (September 30, 2021). Patient response was classified according to the clinical records as complete (total resolution of tumor burden), partial (evidence of a decrease in tumor burden without total resolution), stable (no change in tumor burden), progression (increase in tumor burden), or unknown. Patients were categorized by survival time as follows: short term (<6 months), medium term (6−24 months), and long term (>24 months). Proportions of patient characteristics, treatment regimens, and treatment responses were stratified by survival categories.

### 2.5 Statistical analysis

Descriptive statistics were used for patient, disease, and treatment characteristics. Frequency (n and %) was determined for each categorical variable of interest, and the median and range were determined for age (continuous variable). Standard statistical tests including the Pearson Chi-square and Fisher exact tests for categorical variables and Kruskal-Wallis tests for continuous and non-normally distributed variables were used to test for significant differences in proportions of variables by survival categories. OS probabilities in patients was evaluated with Kaplan-Meier analysis, with the log-rank test used to assess for differences by stratification variables of interest. OS estimates at one, two, and five years and were assessed by sex, ECOG PS, LDH, sodium, and hemoglobin levels, and performance of thoracic RT and PCI. For all statistical tests, a *P*-value of ≤0.05 was considered statistically significant.

Univariable followed by multivariable Cox hazard regression was used to evaluate patient, disease, and treatment characteristics associated with OS. To adjust for the immortality bias associated with having lived long enough to receive thoracic or brain RT, landmarked survival curves were modeled including all patients who survived ≥6 months. Multivariable Cox hazard regression modeling was used to identify patient and disease characteristics and treatment regimens associated with long-term survival.

## 3 Results

### 3.1 Patient demographics

Between 2004 and 2018, a total of 304 patients were identified as having received CT with or without concomitant RT for LS-SCLC and were included in this study. Baseline patient and disease characteristics are summarized in **Table 1**. The population’s mean age was 67 years, and there were more females (57.9%) than males (42.1%). The majority of patients had ECOG PS 0−2 (89.4%) and Stage III disease (67.4%). Long-term survivors represented 39.1% (n = 119) of the overall cohort. They were less likely to have ECOG 3−4 or any abnormal laboratory results (LDH, sodium, or hemoglobin levels) than short- or medium-term survivors. Smoking status had no impact on survival.

**Table 2** presents the pattern of treatment regimens. Significantly more patients received initial cisplatin (75.3%) than carboplatin (24.7%; *P* = 0.005). RT to any site was given to 86.8% of patients and PCI was used in less than 50% of patients. Approximately 19% of patients received no thoracic RT and 16% more received only palliative intention RT. Most patients achieved complete/partial response (72.7%) to the prescribed therapy rather than remaining stable (11.2%) or experiencing initial disease progression (8.9%). CT was completed by 86.2% of patients, including 91.6% of long-term, 90.4% of medium-term, and 41.4% of short-term survivors. CT was delayed for ≥1 cycle in 79.0% of patients and more commonly among medium- (84.0%) and long-term (78.2%) than short-term survivors (55.2%). Complete/partial response was achieved by 79.8%, 73.1%, and 41.4% of long-, medium-, and short-term survivors, respectively. Thoracic RT was administered to 80.6% of patients, including 83.2%, 85.3%, and 44.8% of long-, medium-, and short-term survivors, respectively. Concurrent thoracic RT was the most common delivery (46.5% of those receiving thoracic RT).

**Table 2:** Treatment characteristics by survival.

Characteristic	Short- term survival	Medium- term survival	Long- term survival	*P*-value^a^
**Patients, n (%)** ^b^	29 (9.5)	156 (51.3)	119 (39.1)	
**Response, n (%)**				<0.001
**Complete or partial**	12 (41.4)	114 (73.1)	95 (79.8)	
**Stable**	−^c^	15 (9.6)	19 (16.0)	
**Progression**	−^c^	20 (12.8)	−^c^	
**Chemotherapy, n (%)**				0.005
**Cisplatin**	15 (51.7)	117 (75.0)	97 (81.5)	
**Carboplatin**	14 (48.3)	39 (25.0)	22 (18.5)	
**Chemotherapy setting, n (%)**				0.312
**Inpatient**	−^c^	14 (9.0)	7 (5.9)	
**Outpatient**	−^c^	142 (91.0)	112 (94.1)	
**Thoracic RT delivery, n (%)**				<0.001
**None**	16 (55.2)	23 (14.7)	20 (16.8)	
**Concurrent**	−^c^	53 (34.0)	56 (47.1)	
**Sequential**	−^c^	48 (30.8)	34 (28.6)	
**Palliative**	8 (27.6)	32 (20.5)	9 (7.6)	
**PCI received, n (%)**	−^c^	47 (30.1)	77 (64.7)	<0.001
**Surgical resection, n (%)**	−^c^	6 (3.9)	17 (14.3)	0.005
**Completed chemotherapy,** **n (%)**	12 (41.4)	141 (90.4)	109 (91.6)	<0.001
**Dose reduction, n (%)**	8 (27.6)	41 (26.3)	40 (33.6)	0.403
**Course delayed, n (%)**	16 (55.2)	131 (84.0)	93 (78.2)	0.004
**Any RT received, n (%)**	14 (48.3)	141 (90.4)	109 (91.6)	<0.001

a Fisher exact test P-value;

b unknown data comprise the differences in characteristic subtotals and the group totals;

c patient numbers ≤5 are censored based on requirements from Manitoba Health. PCI, prophylactic cranial irradiation; RT, radiotherapy; Chemotherapy setting is for cycle 1.

### 3.2 Survival analysis

**Figure 1:**
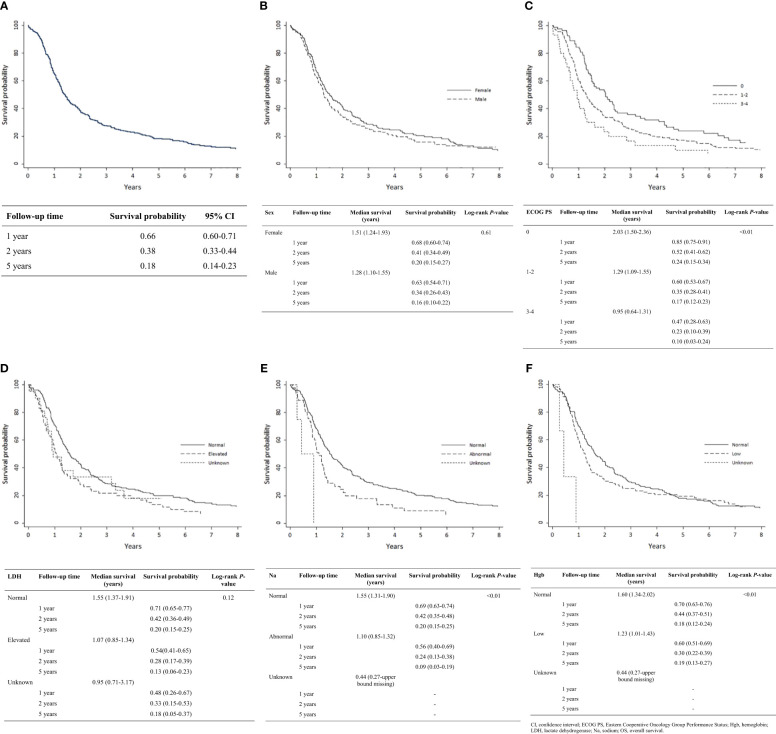
Analysis of OS by patient characteristic: **(A)** Overall cohort (n = 304); **(B)** Sex (n = 304); **(C)** ECOG PS (n = 302); **(D)** LDH (n = 304); **(E)** Serum sodium (n = 304); **(F)** Hemoglobin (n = 304). CI, confidence interval; ECOG PS, Eastern Cooperative Oncology Group Performance Status; Hgb, hemoglobin; LDH, lactate dehydrogenase; Na, sodium; OS, overall survival.

**Figure 2:**
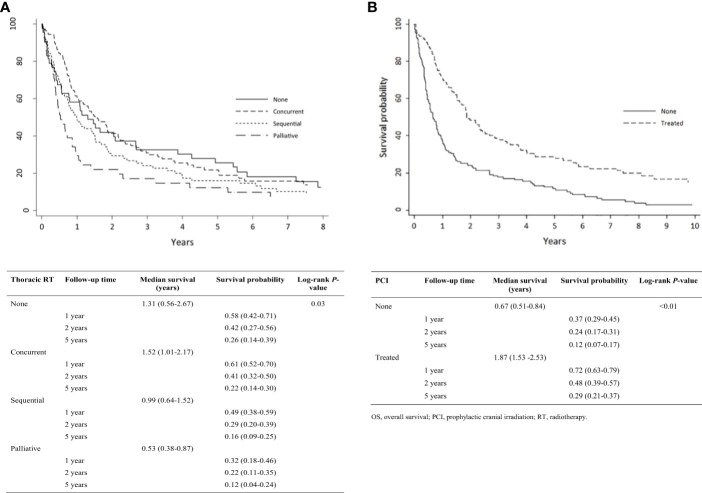
Analysis of limited-stage patient’s OS by treatment pattern who were alive at 0.5 years (N = 275): **(A)** Type of thoracic RT; **(B)** PCI. OS, overall survival; PCI, prophylactic cranial irradiation; RT, radiotherapy.

Kaplan-Meier analysis demonstrated OS estimates at one, two, and five years of 66%, 38%, and 18%, respectively (**Figure 1A**). Females were more likely than males to survive two or five years, but this difference was not statistically significant (*P* = 0.61) (**Figure 1B**). Patients with ECOG PS 0 had significantly higher one-, two-, and five-year survival estimates compared with those with ECOG PS 1-2 and PS 3-4 (*P* < 0.01) (**Figure 1C**). Survival rates were higher among patients with normal vs. elevated LDH levels (>230 U/L) at diagnosis but were not statistically significant (*P* = 0.12) (**Figure 1D**). Survival rates were significantly higher among patients with normal vs. abnormal (<135 or >147 mEq/L) sodium levels (*P* < 0.01; **Figure 1E**) and with normal vs. low hemoglobin levels (males <140 g/L, females <120 g/L) at diagnosis (*P* < 0.01; **Figure 1F**). Receipt of thoracic RT was associated with higher survival rate (*P* < 0.01). Median OS values for concurrent, sequential, and palliative thoracic RT were 1.9, 1.5, and 1.0 years, respectively. Landmarked OS analysis of patients surviving at least 6 months showed a significant difference in OS by type of RT (*P* = 0.03; **Figure 2A**). Patients treated with PCI had a median survival of 2.4 years and experienced higher one-, two-, and five-year survival estimates than patients who did not receive PCI treatment; this pattern of longer survival in patients who received PCI was also seen in the OS analysis landmarked at 6 months (*P* < 0.01; **Figure 2B**).

On univariable hazard regression analysis, OS was significantly associated with ECOG PS, age at diagnosis, receipt of thoracic RT, receipt of PCI, and T-stage. On multivariable hazard regression analysis, T-stage, PCI and ECOG PS were independent predictors of OS after adjusting for other variables in the model (**Table 3**). A landmarked multivariable analysis examining only patients surviving for ≥6 months was performed to account for the immortality bias in patients receiving thoracic RT or PCI and showed significance with only PCI (**Table 4**).

**Table 3:** Multivariable analysis of the full cohort (N = 302).

Univariable						Multivariable			
Variable	Categories	Hazard ratio	95% CI	*P*-value	Overall *P*-value	Hazard ratio	95% CI	*P*-value	Overall *P*-value
**ECOG**	0	Reference	–	–		Reference	–	–	
	1-2	1.43	1.08-1.89	0.011		1.40	1.06-1.87	0.019	
	3-4	2.06	1.33-3.18	0.001	**0.002**	1.44	0.90-2.30	0.128	**0.049**
**Treatment**	Cisplatin (I)	Reference	–	–		n/a			
	Cisplatin (C)	0.78	0.49-1.24	0.3					
	Carboplatin (I)	2.07	0.91-4.71	0.082					
	Carboplatin (C)	0.95	0.57-1.57	0.838	0.081				
**Sex**	Female	Reference	–	–		n/a			
	Male	1.07	0.84-1.36	0.608	0.608				
**Thoracic RT (yes/no)**	No	Reference	–	–		n/a			
	Yes	0.83	0.62-1.12	0.23	0.229				
**Thoracic RT (original)**	None	Reference				Reference	–	–	
	Concurrent	0.70	0.50-0.97	0.035		0.86	0.60-1.23	0.397	
	Sequential	0.85	0.60-1.20	0.351		0.82	0.57-1.19	0.294	
	Palliative	1.29	0.87-1.90	0.205	**0.005**	1.08	0.71-1.64	0.723	0.464
**PCI**	No	Reference	–	–		Reference	–	–	
	Yes	0.44	0.34-0.56	<0.001	**<0.001**	0.49	0.38-0.65	<0.001	**<0.001**
**T Stage**	T0	Reference	–	–		Reference	–	–	
	T1	1.51	0.65-3.54	0.338		1.38	0.58-3.31	0.464	
	T2	1.78	0.78-4.08	0.171		1.75	0.75-4.09	0.196	
	T3	1.78	0.74-4.26	0.197		1.97	0.80-4.82	0.139	
	T4	2.43	1.06-5.57	0.036	**0.024**	2.47	1.07-5.71	0.034	**0.011**
**N Stage**	N0	Reference	–	–		n/a			
	N1	1.09	0.71-1.66	0.707					
	N2	1.43	1.04-1.96	0.027					
	N3	1.50	0.91-2.46	0.11	0.085				
**Age**	Continuous	1.02	1.01-1.04	0.001	**<0.001**	1.01	1.00-1.03	0.083	0.079
**LDH**	Continuous	1.00	1.00-1.00	0.282	0.282	n/a			
**Sodium**	Continuous	0.98	0.95-1.00	0.084	0.084	n/a			
**Hemoglobin**	Continuous	0.99	0.99-1.00	0.212	0.212	n/a			
**Smoking (pack years)**	Continuous	1.01	1.00-1.01	0.115	0.115	n/a			

CI, confidence interval; ECOG PS, Eastern Cooperative Oncology Group Performance Status; LDH, lactate dehydrogenase; HR, hazard ratio; PCI, prophylactic cranial irradiation; T-stage, tumor size; N-stage, nodal status.

P-values <0.05 are bolded. n/a = not applicable.

**Table 4:** Multivariable analysis of the landmarked cohort of patients with LS-SCLC who survived to 6 months (N = 275).

Univariable						Multivariable			
Variable	Categories	Hazard ratio	95% CI	*P*-value	Overall *P*-value	Hazard ratio	95% CI	*P*-value	Overall *P*-value
**ECOG**	0	Reference	–	–		Reference	–	–	
	1 to 2	1.37	1.03-1.82	0.033		1.39	1.04-1.87	0.027	
	3 to 4	1.72	1.06-2.79	0.028	**0.035**	1.31	0.77-2.20	0.316	0.077
**Treatment**	Cisplatin (I)	Reference	–	–		n/a			
	Cisplatin (C)	0.92	0.54-1.55	0.748					
	Carboplatin (I)	1.06	0.51-2.20	0.878					
	Carboplatin (C)	1.01	0.57-1.80	0.964	0.935				
**Sex**	Female	Reference	–	–		n/a			
	Male	1.04	0.80-1.34	0.763	0.763				
**Thoracic RT (yes/no)**	No	Reference	–	–		n/a			
	Yes	1.11	0.79-1.57	0.552	0.552				
**Thoracic RT (original)**	None	Reference	–	–		Reference	–	–	
	Concurrent	0.93	0.63-1.35	0.691		1.09	0.73-1.63	0.674	
	Sequential	1.20	0.81-1.77	0.356		1.15	0.76-1.74	0.497	
	Palliative	1.58	1.01-2.46	0.045	**0.035**	1.34	0.83-2.15	0.227	0.666
**PCI**	No	Reference	–	–		Reference	–	–	
	Yes	0.47	0.37-0.62	<0.001	**<0.001**	0.52	0.39-0.69	<0.001	**<0.001**
**T Stage**	T0	Reference	–	–		Reference	–	–	
	T1	1.42	0.61-3.34	0.418		1.36	0.57-3.28	0.484	
	T2	1.64	0.71-3.77	0.244		1.63	0.69-3.83	0.262	
	T3	1.61	0.66-3.90	0.292		1.73	0.70-4.30	0.235	
	T4	2.28	0.99-5.25	0.052	**0.036**	2.26	0.97-5.24	0.058	0.056
**N Stage**	N0	Reference	–	–		n/a			
	N1	1.15	0.74-1.79	0.546					
	N2	1.45	1.04-2.03	0.028					
	N3	1.43	0.84-2.46	0.192	0.129				
**Age**	Continuous	1.02	1.01-1.04	0.005	**0.005**	1.01	1.00-1.03	0.133	0.133
**LDH**	Continuous	1.00	1.00-1.00	0.708	0.709	n/a			
**Sodium**	Continuous	0.97	0.95-1.00	0.08	**0.080**	n/a			
**Hemoglobin**	Continuous	0.99	0.99-1.00	0.115	0.115	n/a			
**Smoking (pack years)**	Continuous	1.01	1.00-1.01	0.173	0.173	n/a			

CI, confidence interval; ECOG PS, Eastern Cooperative Oncology Group Performance Status; LDH, lactate dehydrogenase; HR, hazard ratio; PCI, prophylactic cranial irradiation; T-stage, tumor size; N-stage, nodal status.

P-values <0.05 are bolded. n/a = not applicable.

## 4 Discussion

This real-world population-based study adds important details to existing knowledge of the demographics, disease characteristics, and treatment outcomes in patients with LS-SCLC treated with CT ± RT. Long-term survivors were younger and more likely to have an ECOG PS of 0 and normal LDH, sodium, and hemoglobin levels at diagnosis. Short-term survivors were less likely to receive cisplatin and more likely to receive palliative RT or no RT. ECOG PS 0 and use of PCI were independently associated with longer OS; of note, PCI remained significant in a landmarked model. ECOG PS was the patient characteristic with the strongest association with survival, providing additional evidence that ECOG PS is an important predictor of survival in patients with lung cancers (1, 34, 39). OS estimates were lower in patients with characteristics traditionally associated with poor prognosis (high LDH, abnormal sodium, and low hemoglobin) (35–38, 40), confirming the results of our previous analysis in the earlier version of this cohort study (34). To our knowledge, this is the most current population-based study to comprehensively evaluate treatment patterns and clinical outcomes in Canadian patients with LS-SCLC treated with CT ± RT.

Findings from this analysis are consistent with those from previous studies. In a real-world study of patients with LS-SCLC and ES-SCLC managed at an Alberta tertiary cancer center, 32.3% of the 65 patients for whom ECOG PS was available had ECOG PS 0, and 63.1% were ECOG 1−2. First-line CT was used in 96.7% of patients with LS-SCLC, including 20.0% as CT alone and 70.8% in combination with RT (32). First-line cisplatin and carboplatin-based regimens were given to 62.1% and 28.4% of patients, respectively. Surgery plus adjuvant therapy was used in 6.7% of patients. Median OS was 40.2 months with first-line surgery plus adjuvant therapy, 32.0 months with first-line CRT, 10.7 months with CT only, CT + thoracic RT, or another first-line therapy (topotecan, cyclophosphamide/doxorubicin/vincristine, capecitabine/temozolomide, or trial agents), and 8.3 months with no treatment. A real-world study from China found that disease stage, good performance status, response to primary systemic treatment, and chemo-irradiation treatments were associated with better OS in patients with LS-SCLC (41). Patients had a median OS of 24.0 months, and the one-, two-, and five-year OS estimates were 78.7%, 48.8%, and 24.2%, respectively. In a retrospective analysis of records from the Ontario Cancer Registry of patients with ES-SCLC and LS-SCLC, the five-year survival rate was 5.8% (42).

Concurrent CRT has been the standard of care for LS-SCLC for three decades, but prognosis remains poor (24). Studies are underway to evaluate the safety and efficacy of novel therapies for LS-SCLC, including ICIs that inhibit programmed death-1 (PD-1) or programmed death ligand 1 (PD-L1) (24, 25). Some of these agents (durvalumab in combination with etoposide and carboplatin or cisplatin and atezolizumab in combination with etoposide and carboplatin) have been approved by Health Canada, the United States Food and Drug Administration, and the European Medicines Agency as first-line therapy for ES-SCLC (43–48). In the LS-SCLC setting, the trial assessing atezolizumab (LU005) includes atezolizumab concurrent with CRT followed by consolidation atezolizumab, while ADRIATIC assesses adding consolidation durvalumab, but does not include it concurrent with CRT (24, 49). Understanding the role of these novel agents in LC-SCLC requires completion of ongoing trials.

While the prevailing standard of care for LS-SCLC remains concurrent CRT, the current analysis further defines which subgroups of patients might survive longest with systemic therapy (2, 9). With the advent of novel therapeutic approaches, identification of molecular subtypes of LS-SCLC may provide information on the susceptibility of certain tumors to different therapies and facilitate clinical decision-making, though this requires additional molecular testing and is not yet a part of routine practice (50). Biomarkers such as PD-L1 and tumor mutation burden (TMB) may have predictive value for ICIs; however, the effect is less certain than in NSCLC (51–55). Neither PD-L1 expression nor TMB status were found to be associated with OS or progression-free survival in patients with ES-SCLC treated with durvalumab or in long-term survival among patients with ES-SCLC treated with atezolizumab (56, 57). Additional research is required on how treatments affect long-term survivors as well as on the way that treatments are delivered to patients with LS-SCLC. For example, treatment before deterioration of the patient’s overall health may prolong survival (58), as could an earlier time to concurrent RT in patients with LS-SCLC and ECOG PS 0−1 (59). In our study, receipt of thoracic RT was associated with longer OS; however, this correlation was not seen when the cohort was landmarked to decrease the immortality bias associated with living long enough to receive both thoracic RT and PCI. Similarly, receipt of PCI was associated with improved survival, which was supported in the landmarked analysis. However, outcomes for patients with LS-SCLC remain poor, highlighting the need for new therapeutic options and ongoing research in this patient population.

PCI use was common in this patient population. It was lower than the rates in some other real-world Canadian studies (50.0%−70.5%) (10, 32, 60). The principal reason for non-use of PCI has been identified as patient refusal due to neurotoxicity concern (14, 60). A landmark randomized trial by Takahashi et al. has also raised concern within the oncologic community that PCI may no longer provide survival benefits in an era where magnetic resonance imaging (MRI) is often used for initial screening (61). While that trial only included patients with ES-SCLC, concern that the benefit of PCI is confined to patients with brain metastases undetectable with computed tomography scanning, but detectable by MRI, also raises questions about benefit in the LS-SCLC population. Recent guidelines still suggest PCI in patients with LS-SCLC who experience a good response to CT+RT (62). Ongoing studies in LS-SCLC and ES-SCLC populations are investigating the use of PCI in conjunction with ICIs (24, 63, 64), and the MAVERICK trial is evaluating the safety and efficacy of MRI brain surveillance with PCI versus MRI brain surveillance alone in patients with SCLC (65).

### 4.1 Study limitations

While observational and retrospective studies are prone to selection bias, our use of a population-based sample of all eligible treated patients in Manitoba is expected to minimize this risk. However, the study cohort was limited to patients who survived long enough to receive treatment with CT, which introduces some selection bias. To adjust for immortality bias, landmarked analyses were performed. As this was a retrospective study, some ECOG PS data were derived from patient description in the medical chart instead of formally stated values, and some laboratory test values were missing in the records analyzed. Smoking pack-year data were missing for 10% of patients. This study did not capture data on dose or timing relative to chemotherapy start for RT administered, although our clinical experience is that it is relatively rare for patients who start thoracic radiotherapy to discontinue it prior to completing the entire course and thus dose heterogeneity is expected to be small and thus non-contributory to survival outcomes in this cohort.

## 5 Conclusions

This study provides supporting evidence that long-term survival in patients with LS-SCLC treated with CT is associated with known prognostic factors such as ECOG PS, laboratory test results, and receipt of treatment in addition to CT. As CT with concurrent RT remains the standard of care for LS-SCLC, a comprehensive understanding of the prognostic factors associated with survival is essential. If trials adding ICIs to the current standard of care prove successful, then this study also provides a baseline real-world estimate of survival for future comparison.

## Data availability statement

The datasets presented in this article are not readily available because the data used in this analysis are owned by the government of Manitoba. The authors were given permission to use the data to conduct the analysis. However, they do not have permission to share the data. The authors did not have special access privileges, and interested researchers would be able to access the data in the same manner as the authors. Requests to access the datasets should be directed to the Provincial Health Research Privacy Committee, Research Manitoba, A201 Chown Building, 753 McDermot Avenue, Winnipeg MB, R3E 0T6 (email: phrpc@researchmb.ca) and CancerCare Manitoba. Instructions can be found at https://www.rithim.ca/phrpc-submission-information and https://www.cancercare.mb.ca/Research/research-office/research-impact-commitee.

## Ethics statement

The studies involving humans were approved by University of Manitoba Health Research Ethics Board (HREB H2015:154 [HS18575]). The studies were conducted in accordance with the local legislation and institutional requirements. Written informed consent for participation was not required from the participants or the participants’ legal guardians/next of kin because in accordance with the national legislation and the institutional requirements.

## Author contributions

DD: first author, trial design, data curation, formal analysis, visualization, writing – review and editing. RR: data acquisition, formal analysis, visualization, writing – review and editing. IS: writing – review and editing. MS: writing – review and editing. DM: writing – review and editing. OB: data curation, formal analysis, writing – review and editing. KG: data curation, formal analysis, writing – review and editing. KR: data acquisition, writing – review and editing. JP: writing – review and editing. CH: writing – review and editing. JK: writing – review and editing. SB: last author, writing – review and editing.

## References

[1] Bahij R Jeppesen SS Olsen KE Halekoh U Holmskov K Hansen O. Outcome of treatment in patients with small cell lung cancer in poor performance status. Acta Oncol (2019) 58:16127. doi: 10.1080/0284186X.2019.1637934 31282251

[2] Jackman DM Johnson BE. Small-cell lung cancer. Lancet (2005) 366:138596. doi: 10.1016/S0140-6736(05)67569-1 16226617

[3] Canadian Cancer Statistics Advisory Committee. Canadian Cancer Statistics: A 2020 special report on lung cancer (2020). Toronto, ON: Canadian Cancer Society (Accessed 27, 2022).

[4] Goldstraw P Chansky K Crowley J Rami-Porta R Asamura H Eberhardt WEE. International association for the study of lung cancer staging and prognostic factors committee, advisory boards, and participating institutions; international association for the study of lung cancer staging and prognostic factors committee advisory boards and participating institutions. The IASLC lung cancer staging project: proposals for revision of the TNM stage groupings in the forthcoming (Eighth) edition of the TNM classification for lung cancer. J Thorac Oncol (2016) 11:3951. doi: 10.1016/j.jtho.2015.09.009

[5] Belluomini L Calvetti L Inno A Pasello G Roca E Vattemi E. SCLC treatment in the immuno-oncology era: current evidence and unmet needs. Front Oncol (2022) 12:840783. doi: 10.3389/fonc.2022.840783 35494084 PMC9047718

[6] Laskin JJ Erridge SC Coldman AJ D yachkova Y Speers C Westeel V. Population-based outcomes for small cell lung cancer: impact of standard management policies in British Columbia. Lung Cancer (2004) 43:716. doi: 10.1016/j.lungcan.2003.07.004 14698532

[7] Lally BE Urbanic JJ Blackstock AW Miller AA Perry MC. Small cell lung cancer: have we made any progress over the last 25 years? Oncologist (2007) 12:1096104. doi: 10.1634/theoncologist.12-9-1096 17914079

[8] Amarasena IU Chatterjee S Walters JA Wood-Baker R Fong KM. Platinum versus non-platinum chemotherapy regimens for small cell lung cancer. Cochrane Database Syst Rev (2015) 2015(8):CD006849. doi: 10.1002/14651858.CD006849.pub3 26233609 PMC7263420

[9] Tartarone A Lerose R Ardito R Troiani L Tedesco B Bozza G. Long-term survival in small cell lung cancer: a case report and review of the literature. Future Oncol (2014) 10:5238. doi: 10.2217/fon.13.213 24754583

[10] BC Cancer. Limited stage disease (2014). Available at: http://www.bccancer.bc.ca/books/lung/management/small-cell-lung-cancer/limited-stage-disease#:~:text=The%20prognosis%20of%20limited%20stage,of%20only%2010%2D12%20weeks (Accessed 12, 2023).

[11] Yan M Toh TS Lindsay PE Weiss J Hueniken K Yeung C. Limited-stage small cell lung cancer: outcomes associated with prophylactic cranial irradiation over a 20-year period at the Princess Margaret Cancer Centre. Clin Transl Rad Oncol (2021) 30:439. doi: 10.1016/j.ctro.2021.06.009 PMC8282904 34296000

[12] Tan Y Yang Q Wu X Zhu H. Curative effect of hyperfractionated accelerated radiotherapy combined with EP chemotherapy regimen on limited-stage small cell lung cancer. J BUON (2021) 26:83743. 34268943

[13] Graabak G Grønberg BH Sandvei MS Nilssen Y Halvorsen TO. Thoracic radiotherapy in limited-stage SCLC-a population-based study of patterns of care in Norway from 2000 until 2018. JTO Clin Res Rep (2021) 3:100270. doi: 10.1016/j.jtocrr.2021.100270 35146461 PMC8801751

[14] Lok BH Ma J Foster A Perez CA Shi W Zhang Z. Factors influencing the utilization of prophylactic cranial irradiation in patients with limited-stage small cell lung cancer. Adv Radiat Oncol (2017) 2:548–54. doi: 10.1016/j.adro.2017.08.001 PMC5707415 29204521

[15] Sun A Durocher-Allen LD Ellis PM Ung YC Goffin JR Ranchandar K. Guideline for the initial management of small cell lung cancer (limited and extensive stage) and the role of thoracic radiotherapy and first-line chemotherapy. Clin Oncol (R Coll Radiol) (2018) 30:658–66. doi: 10.1016/j.clon.2018.06.008 30007803

[16] Lung Cancer Canada. Treatment of small cell lung cancer (2020). Available at: https://www.lungcancerCanada.ca/en-CA/Lung-Cancer/Treatment-Information/Treatment-of-small-cell-lung-cancer.aspx.

[17] Gergen AK Scott CD Mitchell JD. Surgery for limited stage small cell lung cancer. J Thorac Dis (2020) 12:62917. doi: 10.21037/jtd.2020.03.79 PMC7656341 33209467

[18] Ernani V Ganti AK. Surgery for limited-stage small cell lung cancer: ready for prime-time? J Thorac Dis (2017) 9:35768. doi: 10.21037/jtd.2017.09.43 PMC5723782 29268345

[19] Chu X Li S Xia B Chu L Yang X Jianjiao N. Patterns of brain metastasis immediately before prophylactic cranial irradiation (PCI): implications for PCI optimization in limited-stage small cell lung cancer. Radiat Oncol (2019) 14:171. doi: 10.1186/s13014-019-1371-4 31533763 PMC6749639

[20] Rusthoven CG SWOG Cancer Research Network. SWOG S1827 (MAVERICK) Testing whether the use of brain scans alone instead of brain scans plus preventive brain radiation affects lifespan in patients with small cell lung cancer. Available at: https://clinicaltrials.gov/study/NCT04155034#participation-criteria.

[21] Jett JR Schild SE Kesler KA Kalemkerian GP. Treatment of small cell lung cancer: Diagnosis and management of lung cancer, 3rd ed: American College of Chest Physicians evidence-based clinical practice guidelines. Chest (2013) 143:e400S19S. doi: 10.1378/chest.12-2363 23649448

[22] Asai N Ohkuni Y Kaneko N Yamaguchi E Kubo A. Relapsed small cell lung cancer: treatment options and latest developments. Ther Adv Med Oncol (2014) 6:6982. doi: 10.1177/1758834013517413 PMC3932054 24587832

[23] Gong J Salgia R. Managing patients with relapsed small-cell lung cancer. J Oncol Pract (2018) 14:35966. doi: 10.1200/JOP.18.00204 PMC6002253 29894664

[24] Senan S Okamoto I G-w L. Design and rationale for a Phase III, randomized, placebo-controlled trial of durvalumab with or without tremelimumab after concurrent chemoradiotherapy for patients with limited-stage small-cell lung cancer: the ADRIATIC study. Clin Lung Cancer (2020) 21:e848. doi: 10.1016/j.cllc.2019.12.006 31948903

[25] Schlick B Shields MD Marin-Acevedo JA Patel I Pellini B. Immune checkpoint inhibitors and chemoradiation for limited-stage small cell lung cancer. Curr Treat Options Oncol (2022) 23:110420. doi: 10.1007/s11864-022-00989-7 PMC9345799 35716328

[26] Horn L Mansfield AS Szczesna A Havel L Krazowski M Hochmair MJ. IMpower133 Study Group. First-line atezolizumab in extensive-stage small-cell lung cancer. N Engl J Med (2018) 379:22209. doi: 10.1056/NEJMoa1809064 30280641

[27] Goldman JW Dvorkin M Chen T Reinmuth N Hotta K Trukhin D. CASPIAN Investigators. Durvalumab, with or without tremelimumab, plus platinum–etoposide versus platinum–etoposide alone in first-line treatment of extensive-stage small-cell lung cancer (CASPIAN): updated results from a randomised, controlled, open-label, phase 3 trial. Lancet Oncol (2021) 22:5165. doi: 10.1016/S1470-2045(20)30539-8 33285097

[28] Antonia SJ Villegas A Daniel D Vicente D Murakami S Hui R. PACIFIC Investigators; Durvalumab after chemoradiotherapy in stage III non–small-cell lung cancer. N Engl J Med (2017) 377:191929. doi: 10.1056/NEJMoa1709937 28885881

[29] Felip E Altorki N Zhou C. IMpower010 Investigators. Adjuvant atezolizumab after adjuvant chemotherapy in resected stage IB-IIIA non-small-cell lung cancer (IMpower010): a randomised, multicentre, open-label, phase 3 trial. Lancet (2021) 398:134457. doi: 10.1016/S0140-6736(21)02098-5 34555333

[30] Gandhi L Rodríguez-Abreu D Gadgeel S Esteban E Felip E De Angelis F. KEYNOTE-189 Investigators. Pembrolizumab plus chemotherapy in metastatic non–small-cell lung cancer. N Engl J Med (2018) 378:207892. doi: 10.1056/NEJMoa1801005 29658856

[31] Reck M Rodríguez-Abreu D Robinson AG Hui R Csőszi T Fülöp A. Five-year outcomes with pembrolizumab versus chemotherapy for metastatic non–small-cell lung cancer with PD-L1 tumor proportion score ≥50%. J Clin Oncol (2021) 39:233949. doi: 10.1200/JCO.21.00174 PMC8280089 33872070

[32] Elegbede AA Gibson AJ Fu H Dean ML Ezeife DA Lau H. Real-world adherence to guideline-recommended treatment for small cell lung cancer. Am J Clin Oncol (2020) 43:23642. doi: 10.1097/COC.0000000000000657 31842113

[33] Aquin T Banerji S Bucher O Dawe D. P1.12-08 the effect of cisplatin versus carboplatin on cancer outcomes for small cell lung cancer patients in a population-based cohort. J Thorac Oncol (2018) 13:S576. doi: 10.1016/j.jtho.2018.08.843

[34] Rittberg R Green S Aquin T Bucher O Banerji S Dawe DE. Effect of hospitalization during first chemotherapy and performance status on small cell lung cancer outcomes. Clin Lung Cancer (2020) 21:e388e404. doi: 10.1016/j.cllc.2020.02.013 32197856

[35] Liu J Wu D Shen B Chen M Zhou X Zhang P. Serum lactate dehydrogenase predicts brain metastasis and survival in limited-stage small cell lung cancer patients treated with thoracic radiotherapy and prophylactic cranial irradiation. Strahlenther Onkol (2022) 198:1094104. doi: 10.1007/s00066-022-01977-4 35857072

[36] Marroncini G Anceschi C Naldi L Fibbi B Baldanzi F Martinelli S. Low sodium and tolvaptan have opposite effects in human small cell lung cancer cells. Mol Cell Endocrinol (2021) 537:111419. doi: 10.1016/j.mce.2021.111419 34389446

[37] Zhang JQ Wang YY Xu KP Qi J Wang X Xu LM. Prognostic evaluation of nutritional indicators in patients with limited-stage small cell lung cancer. Zhonghua Zhong Liu Za Zhi (2019) 41:93742. doi: 10.3760/cma.j.issn.0253-3766.2019.12.010 31874552

[38] Bernhardt D Aufderstrasse S König L Adeberg S Bozorgmehr F Christopoulos P. Impact of inflammatory markers on survival in patients with limited disease small-cell lung cancer undergoing chemoradiotherapy. Cancer Manag Res (2018) 10:65639. doi: 10.2147/cmar.s180990"10.2147/CMAR.S180990 PMC6280890 30555261

[39] Crawley D Beckmann K Ravindra S Josephs DH Spicer JF Montes A. Association of baseline characteristics and survival in patients with advanced non-small cell lung cancer (NSCLC) treated with immune checkpoint inhibitors (CPIs): Real-world evidence. J Clin Oncol (2020) 38(15_suppl):e21625. doi: 10.1200/JCO.2020.38.15_suppl.e21625

[40] Bremnes RM Sundstrom S Aasebø U Kaasa S Hatlevoll R Aamdal S. Norweigian Lung Cancer Study Group. The value of prognostic factors in small cell lung cancer: results from a randomised multicenter study with minimum 5 year follow-up. Lung Cancer (2003) 39(3):30313. doi: 10.1200/JCO.2020.38.15_suppl.e21625 12609569

[41] Ma X Zhang Z Chen X Zhang J Nie J Da L. Prognostic factor analysis of patients with small cell lung cancer: real-world data from 988 patients. Thorac Cancer (2021) 12:184150. doi: 10.1111/1759-7714.13846 PMC8201544 33955685

[42] Doherty J Dawe DE Pond GR Ellis PM. The effect of age on referral to an oncologist and receipt of chemotherapy among small cell lung cancer patients in Ontario, Canada. J Geriatr Oncol (2019) 10:44958. doi: 10.1016/j.jgo.2018.10.001 30318328

[43] AstraZeneca Canada Inc. IMFINZI® (durvalumab for injection) Product Monograph. AstraZeneca Canada Inc., Mississauga, Ontario, Canada (2022).

[44] AstraZeneca Inc. IMFINZI^®^ (durvalumab) Prescribing Information. AstraZeneca Pharmaceuticals LP Wilmington, Delaware, United States (2021).

[45] AstraZeneca AB. IMFINZI^®^ (durvalumab) Summary of Product Characteristics. AstraZeneca AB, Södertälje, Sweden (2022).

[46] Hoffmann-La Roche Limited. TECENTRIQ^®^ (atezolizumab for injection) Product Monograph. Hoffmann-La Roche Ltd., Mississauga, Ontario, Canada (2022).

[47] Genentech Inc. TECENTRIQ^®^ (atezolizumab) Product Information. Genentech, Inc., South San Francisco, California, United States (2021).

[48] Roche Registration GmbH. TECENTRIQ^®^ (atezolizumab) Summary of Product Characteristics. Roche Registration GmbH. Grenzach-Wyhlen, Germany (2022).

[49] Ross HJ Hu C Higgins KA Jabbour SA Kozono DE Owonikoko TK. NRG Oncology/Alliance LU005: A phase II/III randomized clinical trial of chemoradiation versus chemoradiation plus atezolizumab in limited stage small cell lung cancer. J Clin Oncol (2020) 38:TPS9082. doi: 10.1200/JCO.2020.38.15_suppl.TPS9082

[50] Rudin CM Poirier JT Byers LA Dive C Dowlati A George J. Molecular subtypes of small cell lung cancer: a synthesis of human and mouse model data. Nat Rev Cancer (2019) 19:289297. doi: 10.1038/s41568-019-0133-9 PMC6538259 30926931

[51] Ott PA Bang YJ Piha-Paul SA Razak ARA Bennouna J Soria JC. T-cell-inflamed gene-expression profile, programmed death ligand 1 expression, and tumor mutational burden predict efficacy in patients treated with pembrolizumab across 20 cancers: KEYNOTE-028. J Clin Oncol (2019) 37:318–27. doi: 10.1200/JCO.2018.78.2276 30557521

[52] Ready N Hellmann MD Awad MM Otterson GA Gutierrez M Gainor JF. First-line nivolumab plus ipilimumab in advanced non-small-cell lung cancer (CheckMate 568): outcomes by programmed death ligand 1 and tumor mutational burden as biomarkers. J Clin Oncol (2019) 37:992–1000. doi: 10.1200/JCO.18.01042 30785829 PMC6494267

[53] Mandal R Samstein RM Lee KW Havel JJ Wang H Krishna C. Genetic diversity of tumors with mismatch repair deficiency influences anti-PD-1 immunotherapy response. Science (2019) 364:485–91. doi: 10.1126/science.aau0447 PMC6685207 31048490

[54] Gandhi L Rodríguez-Abreu D Gadgeel S Esteban E Felip E De Angelis F. KEYNOTE-189 Investigators. Pembrolizumab plus chemotherapy in metastatic non-small-cell lung cancer. N Engl J Med (2018) 378:207892. doi: 10.1056/NEJMoa1801005 29658856

[55] Lu Y Zhang X Ning J Zhang M. Immune checkpoint inhibitors as first-line therapy for non-small cell lung cancer : a systematic evaluation and meta-analysis. Hum Vaccine Immunother (2023) 19:2169531. doi: 10.1080/21645515.2023.2169531 PMC10038046 36715018

[56] Paz-Ares L Goldman JW Garassino MC Dvorkin M Trukhin D Statsenko G. PD-L1 expression, patterns of progression and patient-reported outcomes (PROs) with durvalumab plus platinum-etoposide in ES-SCLC: results from CASPIAN. Ann Oncol (2019) 30(Suppl. 5):v928–9. doi: 10.1093/annonc/mdz394

[57] Liu SV Horn L Mok T Mansfield A De Boer R Losonczy G. IMpower133: Characterisation of long-term survivors treated first-line with chemotherapy ± atezolizumab in extensive-stage small cell lung cancer. Ann Oncol (2020) 31:S10323. doi: 10.1016/j.annonc.2020.08.1543

[58] Gressner O Popp H Mey U Friedrichs N Strehl J Sauerbruch T. Long-term survival of a patient with small cell lung cancer after nine lines of chemotherapy and radiation. Onkologie (2008) 31:46972. doi: 10.1159/000142396 18787355

[59] Jones GS Khakwani A Pascoe A Foweraker K McKeever TM Hubbard RB. Factors associated with survival in small cell lung cancer: an analysis of real-world national audit, chemotherapy and radiotherapy data. Ann Palliat Med (2021) 10:405568. doi: 10.21037/apm-20-1824 33894719

[60] Giuliani M Sun A Bezjak A Ma C Le LW Brade A. Utilization of prophylactic cranial irradiation in patients with limited stage small cell lung carcinoma. Cancer (2010) 116:56949. doi: 10.1002/cncr.25341 20803612

[61] Takahashi T Yamanaka T Seto T Harada H Nokihara H Saka H. Prophylactic cranial irradiation versus observation in patients with extensive-disease small-cell lung cancer: a multicentre, randomised, open-label, phase 3 trial. Lancet Oncol (2017) 18:66371. doi: 10.1016/S1470-2045(17)30230-9 28343976

[62] Simone CB 2nd Bogart JA Cabrera AR. Radiation therapy for small cell lung cancer: an ASTRO clinical practice guideline. Pract Radiat Oncol (2020) 10:15873. doi: 10.1016/j.prro.2020.02.009 PMC10915746 32222430

[63] Reinmuth N DeMarinis F Leighl N Sadow S Davey K Özgüroğlu M. EP14.05-009 LUMINANCE: a Phase IIIb study of durvalumab + platinum-etoposide for first-line treatment of extensive-stage SCLC (ES-SCLC). J Thorac Oncol (2022) 17(Suppl):S547. doi: 10.1016/j.jtho.2022.07.984

[64] Ross HJ Hu C Higgins KA Jabbour S Kozono D Owonikok T. P48.02 NRG Oncology/Alliance LU005: chemoradiation vs. chemoradiation plus atezolizumab in limited stage small cell lung cancer. J Thorac Oncol (2021) 16(suppl):S499S500. doi: 10.1016/j.jtho.2021.01.872 PMC13504423 42532339

[65] Rusthoven C. MAVERICK: MRI Brain Surveillance Alone versus MRI Surveillance and Prophylactic Cranial Irradiation: A Randomized Phase III Trial in Small-Cell Lung Cancer (MAVERICK). Available at: https://www.swog.org/sites/default/files/docs/2021-02/S1827-Maverick%20Trial.pdf.

